# Nicotinamide mononucleotide improves spermatogenic function in streptozotocin-induced diabetic mice via modulating the glycolysis pathway: Nicotinamide mononucleotide improves spermatogenic function

**DOI:** 10.3724/abbs.2022099

**Published:** 2022-08-04

**Authors:** Duo Ma, Linlin Hu, Jinyuan Wang, Min Luo, Aihong Liang, Xiaocan Lei, Biyun Liao, Meixiang Li, Ming Xie, Haicheng Li, Yiwei Gong, Dan Zi, Xiangrun Li, Xi Chen, Xucai Liao

**Affiliations:** 1 Hunan Province Collaborative Innovation Base of Endocrinology & Metabolism Science and Education for Postgraduates The First Affiliated Hospital of Shaoyang University and Hengyang Medical School University of South China Hengyang 422000 China; 2 Institute of Clinical Anatomy & Reproductive Medicine Hengyang Medical School University of South China Hengyang 421001 China; 3 Reproductive Medicine Center The Affiliated Hospital of Youjiang Medical University for Nationalities Baise 533000 China

**Keywords:** nicotinamide mononucleotide, diabetes, spermatogenic function, Sertoli cell, glycolysis

## Abstract

Spermatogenic dysfunction is one of the major secondary complications of diabetes; however, the underlying mechanisms remain ill-defined, and there is no available drug or strategy for the radical treatment of diabetic spermatogenic dysfunction. Therefore, the objective of this study is to investigate the protective effects of nicotinamide mononucleotide (NMN) on testicular spermatogenic function in streptozotocin (STZ)-induced diabetic mice. The results show that oral administration of NMN significantly increases the body and testis weight and the number of sperms. Moreover, the abnormal sperm count and the rate of sperm malformation are significantly decreased compared with the saline-treated diabetic mice. Histological analysis reveals that NMN treatment significantly increases the area and diameter of seminiferous tubules, accompanied by an increased number of spermatogenic cells and sperms. Immunohistochemistry and qRT-PCR results show that NMN increases Bcl-2 expression and decreases Bax expression in the testis. NMN also increases the protein expression of Vimentin and the mRNA expressions of *WT1* and *GATA4*. In addition, qRT-PCR, western blot analysis and immunohistochemistry results also show that NMN increases the expressions of glycolysis-related rate-limiting enzymes including HK2, PKM2, and LDHA. In summary, this study demonstrates the protective effects of NMN on the testis in an STZ-induced diabetic mice model. NMN exerts its protective effects via reducing spermatogenic cell apoptosis by regulating glycolysis of Sertoli cells in diabetic mice. This study provides an experimental basis for the future clinical application of NMN in diabetes-induced spermatogenic dysfunction.

## Introduction

Diabetes mellitus (DM) is a metabolic disorder manifested by chronic hyperglycemia with potentially severe effects on insulin sensitivity and secretion [1]. According to the reports of International Diabetes Federation, the number of diabetic patients will increase to 693 million in 2045, accounting for about 10% of the world’s population [2]. DM affects many body systems and is frequently accompanied by many complications, including nephropathy, neuropathy, and retinopathy. DM is also associated with a high risk of cardiovascular diseases and male infertility [3]. Epidemiological studies showed that about 50% of diabetic patients suffer from different degrees of reproductive diseases, such as decreased libido and impotence [4]. In addition, diabetes can impair spermatogenesis [5], destroy testicular tissue structure, and reduce sperm quality, leading to infertility [6]. In the testes, glucose metabolism is pivotal for spermatogenesis. Sertoli cells (SCs) are well known for their ability to produce lactate at a high rate; lactate and pyruvate are consumed by pachytene spermatocytes and round spermatids [7]. It has been shown that in the testis of diabetic rats, lactate dehydrogenase (LDH) activity is significantly reduced, resulting in glycogen accumulation and reduced lactate production [8]. Therefore, abnormal glycolysis is closely related to spermatogenic disorder in diabetes, which may provide novel targets for treating this disease.

Nicotinamide mononucleotide (NMN) is synthesized from nicotinamide and 5′-phosphoribosyl-1-pyrophosphate (PRPP) by nicotinamide phosphoribosyl transferase (NAMPT), the rate-limiting NAD ^+^ biosynthetic enzyme in mammals [ 9, 10] . Meanwhile, previous studies have shown that NMN is also present in vegetables, fruits and meat [11]. Systemic NMN administration effectively enhances NAD ^+^ biosynthesis in various peripheral tissues, including the liver [12], kidney [13], and testis [9]. NMN supplementation could affect diverse diseases, including myocardial, Alzheimer’s disease, and diabetes [14]. Revollo *et al*. [15] and Ramisey *et al*. [16] showed that NMN ameliorates impairments in glucose-stimulated insulin secretion in aged wild-type mice and genetic mouse models. NMN treatment also significantly improves both insulin action and secretion in diet- and age-induced type 2 diabetic or obese mouse models [17]. However, the specific mechanism of NMN in diabetes treatment is unclear. NAD ^+^ plays an important role in glycolysis, and LDH converts pyruvate to lactate and reduces NADH to NAD ^+^ [ 18, 19] . Therefore, we speculate that NMN may improve the spermatogenic dysfunction in diabetes by regulating the glycolysis pathway.

In the present study, we verified the effect of NMN on diabetic testicular dysfunction by detecting some factors including Bcl-2-associated X protein (Bax), B-cell lymphoma-2 (Bcl-2), Vimentin, Wilms’ tumor gene 1 (WT1), GATA4, hexokin ase 2 (HK2), pyruvate kinase muscle isoform 2 (PKM2), and lactate dehydrogenase A (LDHA). Our findings provide a new idea for treating diabetic testicular dysfunction.

## Materials and Methods

### Experimental animals

**Figure 1:**
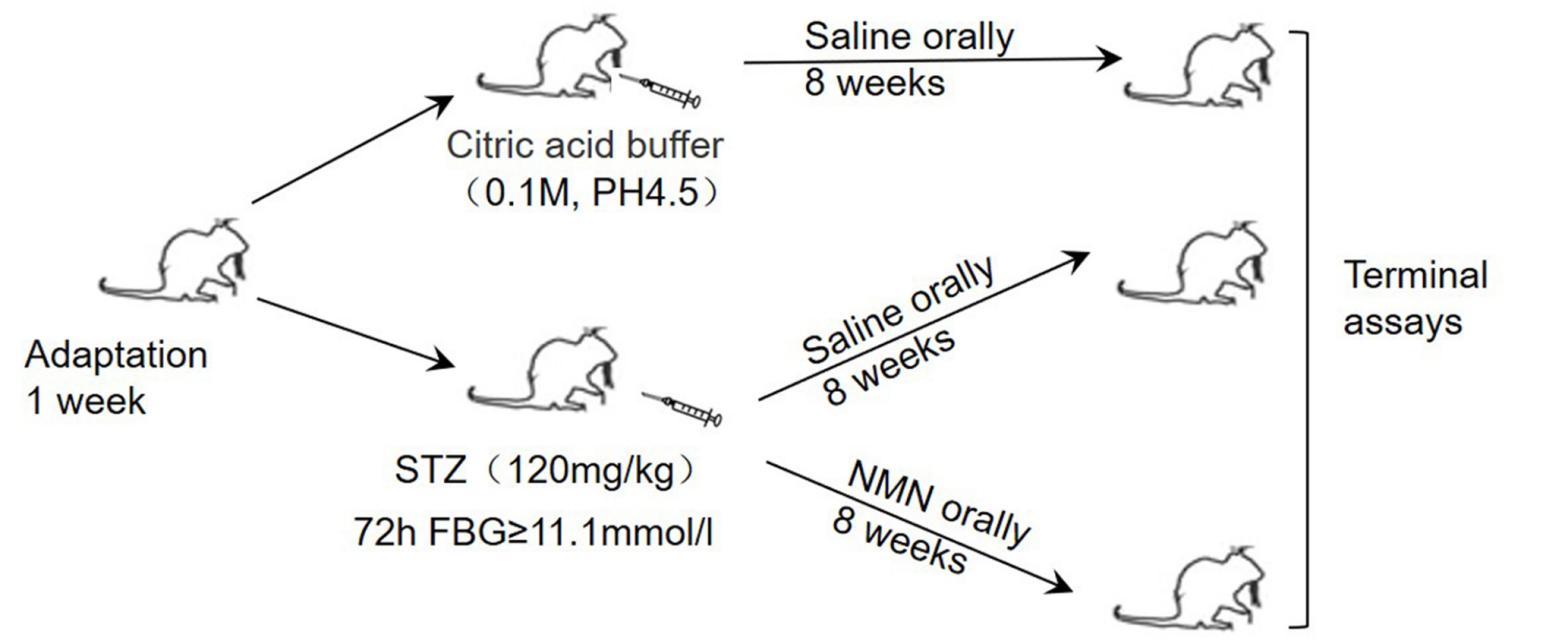
Figure 1 The overall experimental design including animal groups and treatments

Male C57BL/6J mice (body weight 19±1 g) were purchased from Hunan SJA Lab Animal Center of Changsha (Changsha, China) with the license number SCXK (Xiang) 2019-0004. The Animal Ethics Committee of the University of South China approved all experimental procedures (No. USC2020031602). All animals were maintained at 23±2°C (humidity, 45%–55%) with a 12/12 h light/dark cycle and given a standard diet (3.1 kcal, 14% protein). After 1 week of adaptive feeding, all mice were randomly divided into the control group (Ctrl group, *n*=8) and diabetic group (model group, *n*=16). Mice were rendered diabetic by a single-dose intraperitoneal injection of STZ (120 mg/kg body weight; S-0130; Sigma, St Louis, USA) dissolved in 0.1 M citrate buffer at pH 4.5. At the same time, the Ctrl group was given an equal volume of citric acid buffer. After 72 h, mice with a fasting blood glucose (FBG) level over 11.1 mM was considered as diabetic [20]. No mice died during the experiment. Diabetic mice were further randomly divided into two groups: DM+saline group (DM group, *n*=8) and DM+NMN group ( *n*=8). In the DM+NMN group, NMN (Shenzhen Hygieia Biotechnology, Shenzhen, China) was administrated orally at 500 mg/kg/day in saline [11], while the Ctrl group and DM group mice were given an equal volume of saline. After 8 weeks of treatment, mice were sacrificed, and relevant tissues were collected for further analysis ( Figure 1).

### Assessment of sperm number

The cauda epididymis of the mice was snipped and placed in 1.5 mL physiological saline at 37°C for 20 min. The sperm suspension (100 μL) was diluted with saline (900 μL), and then the diluted semen was aspirated and dropped into a blood cell counting plate using a micropipette. The sperm concentration in the five central squares was counted, and the average was multiplied by 10 ^7^ to obtain the number of sperm per liter of semen and the number of abnormal sperm.

### Histopathologic analysis and the area and diameter of seminiferous tubules analysis

The left testis and the epididymis were dissected and fixed in 4% paraformaldehyde overnight at room temperature, embedded in paraffin, sectioned into 4-μm-thick slices, deparaffinized, and stained with hematoxylin and eosin. The morphology of the testes and the number of spermatogonia and spermatocytes were evaluated under a light microscope. Then, the diameter and area of seminiferous tubules were obtained by random selection of 50 cross sections of the seminiferous tubules from each mouse.

### Immunohistochemical analysis

The 4-μm slices were sectioned. The sections were permeabilized with 1% Triton X-100 in phosphate-buffered saline (PBS) for 30 min at room temperature, boiled in 100 mM sodium citrate (pH 6.0) three times for 6 min each at 5-min intervals for antigen retrieval. The sections were then incubated with 3% hydrogen peroxide for 30 min to remove endogenous peroxidase, followed by blocking in 5% bovine serum albumin at room temperature for 1 h. The sections were then incubated overnight at 4°C with primary goat polyclonal antibodies against Vimentin (1:300 dilution; Bio World Technology, Nanjing, China), Bax (1:100 dilution; Santa Cruz Biotechnology, Oregon, USA), Bcl-2 (1:100 dilution; Santa Cruz Biotechnology), HK2 (1:400 dilution; Cell Signaling Technology, Danvers, USA), PKM2 (1:500 dilution; Cell Signaling Technology), and LDHA (1:300 dilution; Cell Signaling Technology) in blocking solution. After three washes with 0.1% Tween-20 in PBS, the samples were incubated with biotin-SP-conjugated rabbit anti-goat IgG secondary antibody (1:100; SA00004–4; Protein Tech Group Inc., Rosemont, USA) in blocking solution at room temperature for 45 min. The stained Vimentin, Bax, Bcl-2, HK2, PKM2, and LDHA proteins were visualized using the 3,3-diaminobenzidine chromogen. Normal goat IgG was used as a negative control. The stained sections were evaluated under a light microscope. Finally, the sections were sealed with neutral resin and observed under an optical microscope. The immunohistochemical positive cells were analyzed by counting the amounts of the positive cells in 50 randomly selected non-overlapping visual fields (magnification, ×400) from each testis section.

### Quantitative real-time polymerase chain reaction (qRT-PCR)

Total RNA was extracted from frozen testicular tissue using Trizol reagent (Invitrogen, Carlsbad, USA). The purity and concentration of total RNA were determined by A260/A280. The cDNA synthesis was performed using the Prime Script 1st strand cDNA Synthesis kit (TaKaRa, Dalian, China) according to the manufacturer’s protocol. Real-time PCR analyses for the gene expression level were performed on the Applied Biosystems 7500 Real-time PCR System (Foster City, USA). The conditions were as follows: after initial incubations at 95°C for 10 min, 40 cycles of amplification were carried out at 95°C for 10 s and 60°C for 30 s. *GAPDH* was used as the reference control, and gene expression levels were calculated using the comparative Ct method. The primer sequences are shown in Table 1.

**Table:** **Table 1** Sequence of primers used for qRT-PCR analysis

Gene	Primer sequence (5′→3′)	Accession No.
*Bcl-2*	F: GGTGGTGGAGGAACTCTTCA	NM_177410.2
	R: ATGCCGGTTCAGGTACTCAG	
*Bax*	F: TGCAGAGGATGATTGCTGAC	NM_177410.2
	R: GATCAGCTCGGGCACTTTAG	
*WT1*	F: ATCCGCAACCAAGGATACAG	DQ537939.1
	R: GGTCCTCGTGTTTGAAGGAA	
*GATA4*	F: ATGCCTGTGGCCTCTATCAC	AF179424.1
	R: GGTGGTGGTAGTCTGGCAGT	
*HK2*	F: CGTGGTAAATGACACAGTTG	BC054472.1
	R: AGTTCCACATTACGCATCTC	
*PKM2*	F: CAGTACAGAATACACACCCA	BC094663.1
	R: GTCATGTCTTATGTGTGGGT	
*LDHA*	F: ACTGTGTAACTGCGAACTCC	BC094019.1
	R: GGGAATGATGAACTTGAAGA	
*GAPDH*	F: ATTGTCAGCAATGCATCCTG	GU214026.1
	R: ATGGACTGTGGTCATGAGCC	

F, forward primer; R, reverse primer.

### Western blot analysis

Frozen testicular tissue was pulverized and homogenized in cold lysis buffer. The homogenate was centrifuged at 12,000 *g* for 20 min at 4°C, and the supernatant was collected. The protein concentration of the supernatant was measured using the BCA Protein Assay kit (CWBIO, Beijing, China). After being boiled at 100°C for 10 min, samples (50 μg protein/lane) were subject to 10% polyacrylamide gel electrophoresis and then transferred to polyvinylidene difluoride (PVDF) membranes (Millipore, Billerica, USA). The membranes were blocked in a 5% non-fat milk in PBST for 2 h at room temperature and then incubated with primary antibodies against HK2 (1:1000 dilution; Cell Signaling Technology), PKM2 (1:1000 dilution; Cell Signaling Technology), LDHA (1:1000 dilution; Cell Signaling Technology), and β-tubulin (1:3000 dilution; Proteintech Group, Inc.) overnight at 4°C. The membranes were washed with PBST and then incubated with HRP-conjugated goat anti-mouse IgG (H+L) secondary antibody (1:5000 dilution; Protein Tech Group Inc) or HRP-conjugated goat anti-rabbit IgG (H+L) secondary antibody (1:5000 dilution; Protein Tech Group Inc.) for 2 h at room temperature. Finally, eECL (CW0049M; CWBIO, Beijing, China) was added, and the Tanon-5500 Chemiluminescence Imaging System (Beijing, China) was used to detect the chemiluminescence of protein bands.

### Statistical analysis

Analysis was performed by one-way analysis of variance (ANOVA). The data are presented as the mean±standard deviation. *P*<0.05 was considered to be statistically significant.

## Results

### Effect of NMN treatment on body weight, testis weight, sperm number, and sperm abnormality in diabetic mice

**Figure 2:**
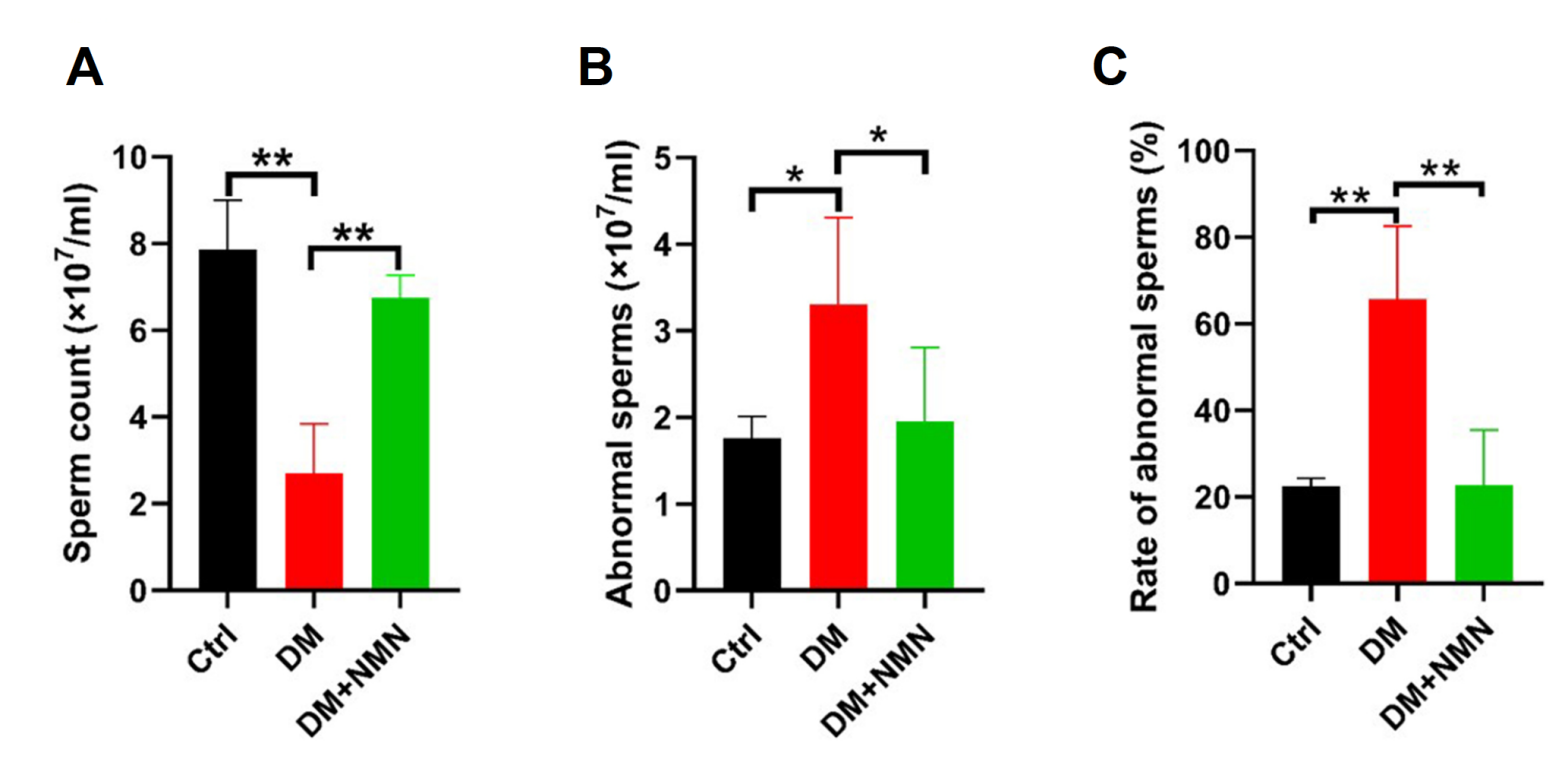
Figure 2 Effect of NMN treatment on sperm parameters in diabetic mice The effect of NMN on sperm count (A), abnormal sperms (B), and the rate of abnormal sperms (C) in testis tissue of diabetic mice. n=8. * P<0.05 and ** P<0.01.

At the beginning of the experiment, all the mice did not differ in body weight (data not shown). As demonstrated in Table 2, after successful induction of diabetes in mice with STZ, the body weight of model mice was significantly lower than that of the Ctrl group. After 8 weeks of NMN treatment, the body weight of DM mice was significantly increased compared with that of untreated DM mice. Similarly, the saline-treated diabetic mice had lower testis weight, and administration of NMN showed an increase in testicular weight compared with the DM group. Regarding the sperm parameters, the number of sperms in saline-treated diabetic mice was significantly decreased and the proportion of abnormal sperms was increased. These results suggested that NMN treatment significantly increased sperm numbers of the diabetic mice and decreased the rate of sperm deformity, which enhanced the reproductive potential of diabetic mice ( Figure 2).

**Table:** **Table 2** Effect of NMN on the body weight, testis weight and sperm parameters in diabetic mice

Group	Body weight (g)	Testis weight (mg)	Sperm count (×10 ^7^)	Abnormal sperm Number (×10 ^7^)	Abnormal sperm rate (%)
	Pre-treatment	After treatment	
Control	21.34±0.83	23.72±2.73	164.54±12.61	7.87±1.13	1.76±0.25	22.44±1.83
DM	19.48±0.75**	15.64±1.97**	138.45±20.40*	2.70±1.14**	3.30±1.01*	65.76±16.88**
DM+NMN	19.46±0.67**	20.52±0.82 ^##^	166.20±4.30 ^#^	6.75±0.53 ^##^	1.95±0.86 ^#^	22.69±12.80 ^##^

* *P*<0.05, ** *P*<0.01 vs control; ^#^ *P*< 0.05, ^##^ *P*<0.01 vs DM.

### Effect of NMN treatment on the epididymis and testicular morphology, diameter, and area of spermatogenic tubules in diabetic mice

**Figure 3:**
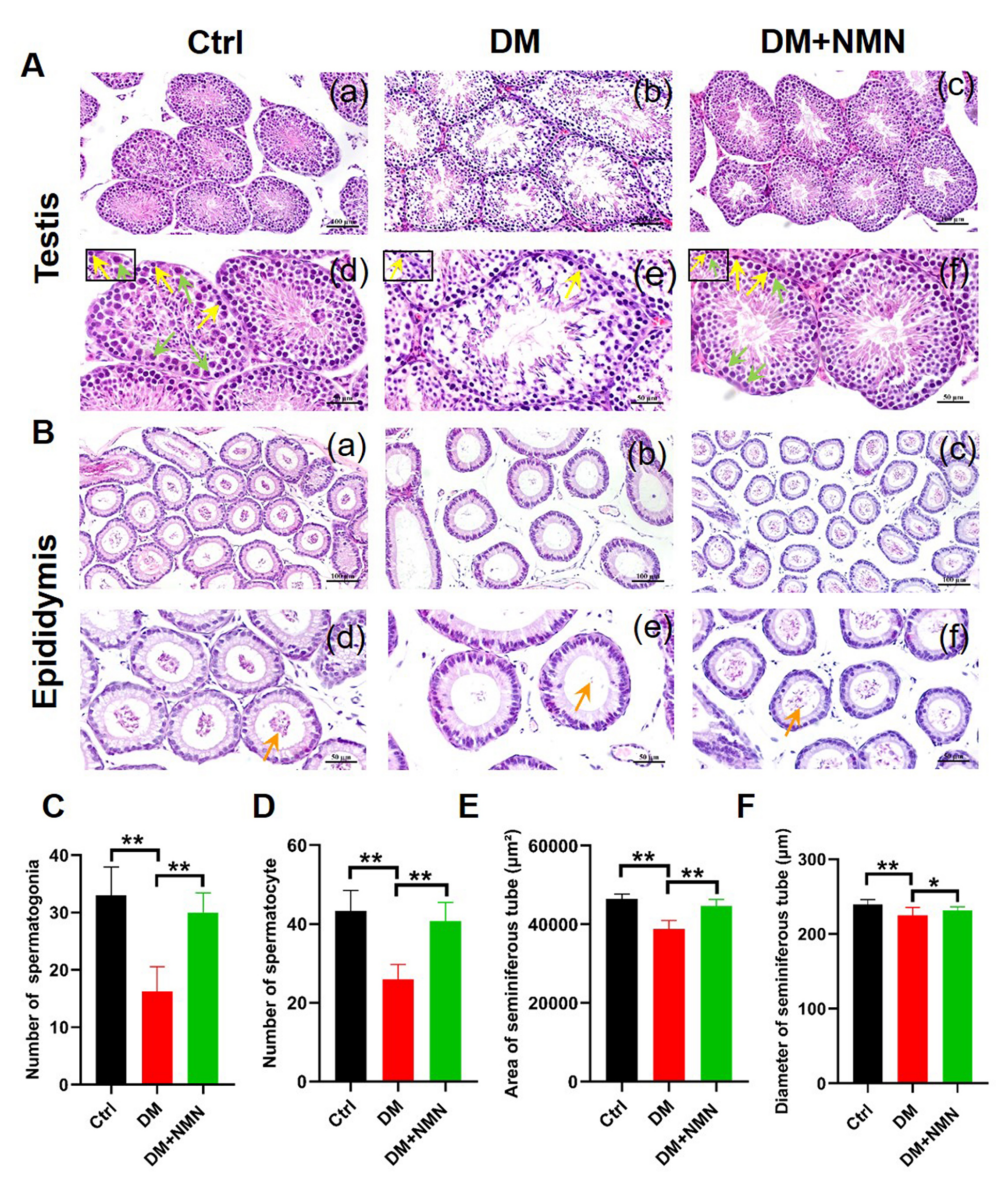
Figure 3 Effect of NMN treatment on the diabetic mice testicular and epididymal histopathology (A,B) Hematoxylin and eosin staining of testicular and epididymal tissue. Scale bars in (a–c) are 100 μm and in (d–f) are 50 μm. (C,D) Quantification of spermatogonia and spermatocytes in testicular tissues. (E,F) The area and diameter of the seminiferous tube in testis. Yellow arrows denote spermatogonia, green arrows denote spermatocytes, and orange arrows denote sperms. n=8. * P<0.05 and ** P<0.01.

Testicular and epididymis histopathological changes of the three groups are presented in Figure 3. In the Ctrl group, the shape of seminiferous tubules was regular, and the spermatogenic cells of the seminiferous epithelium at all levels were arranged neatly, mostly in 6–7 layers, and many spermatozoa were present in the lumen of seminiferous tubules ( Figure 3A, a,d). Furthermore, epididymal sections showed many sperms in the lumen of epididymal tubules ( Figure 3B a,d). In contrast, the number of spermatogenic epithelial cell layers in saline-treated diabetic mice testis was significantly decreased; most of them were 3–4 layers or less, and the number of sperms was decreased, and the spermatogenic function was weakened ( Figure 3A, b,e). Quantification of the spermatogonia and spermatocytes showed that the saline-treated diabetic mice had a lower number of spermatogonia and spermatocytes than the Ctrl group mice ( Figure 3C,D). Meanwhile, the mean area and diameter of seminiferous tubules in diabetic mice were smaller than these in Ctrl mice ( Figure 3E,F). In addition, a few sperms were found in the epididymal lumen compared with the Ctrl group ( Figure 3B, b,e). Interestingly, the spermatogenic epithelium damage in the NMN group was significantly alleviated, and the number of spermatogenic cell layers, spermatogonia, spermatocytes, and sperms were increased. Simultaneously, the area and diameter of spermatogenic tubules were increased, which led to the recovery of spermatogenesis to some extent ( Figure 3).

### Effect of NMN on apoptosis in testicular tissues from diabetic mice

**Figure 4:**
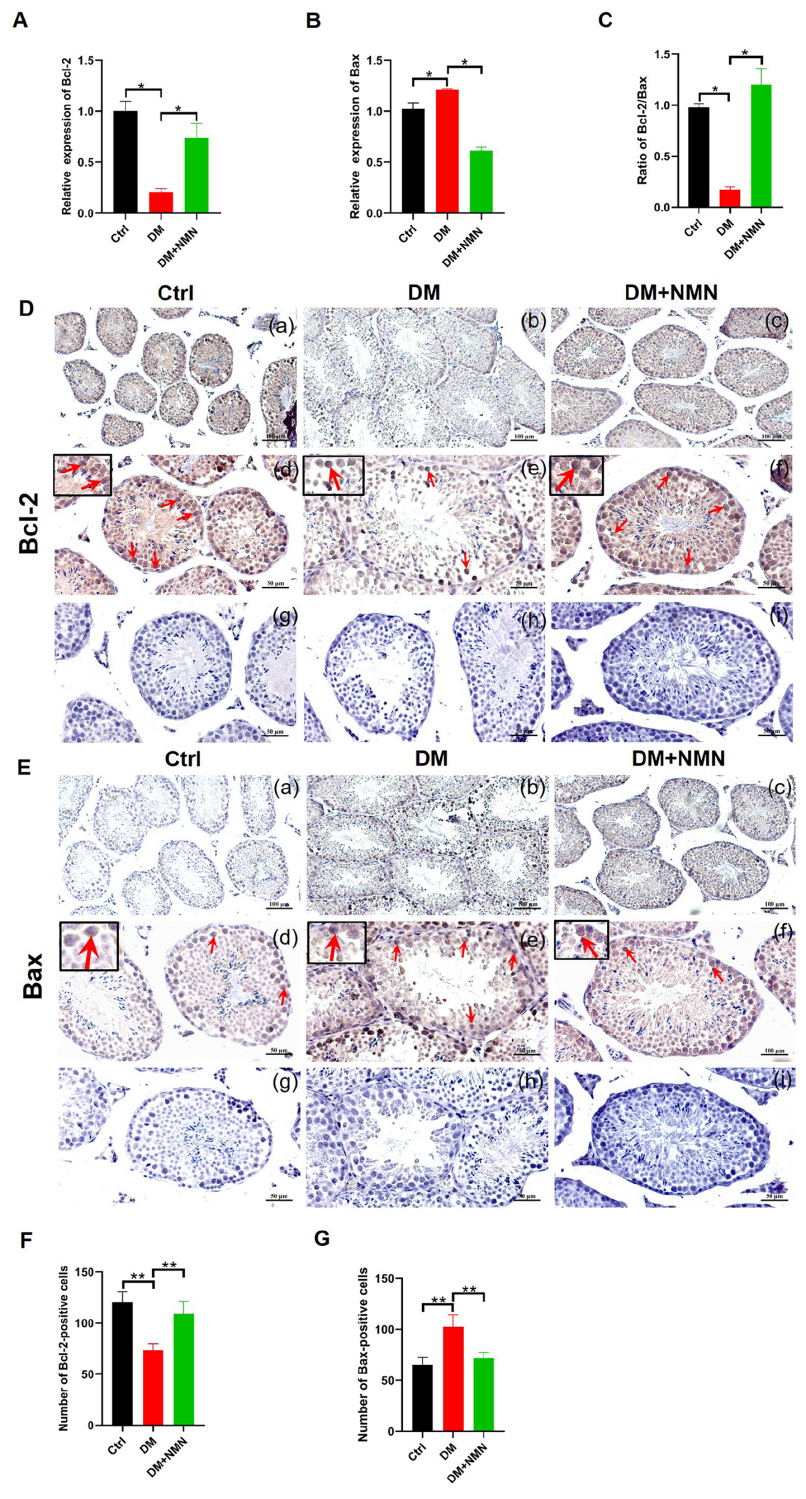
Figure 4 Effect of NMN on apoptosis in testicular tissues of diabetic mice (A,B) qRT-PCR analysis of Bcl-2 and Bax mRNA expressions in testis. (C) Ratio analysis of Bcl-2/ Bax expression. n=4. (D,E) Immunohistochemistry analysis of Bcl-2 and Bax expressions in testis. Scale bars in (a–c) are 100 μm and in (d–i) are 50 μm. (F,G) Bcl-2 and Bax positive cell count. The red arrows indicate the cells with positive signals. n=8. * P<0.05 and ** P<0.01.

The expression levels of apoptosis-related genes, including *Bcl-2* and *Bax* in mice testis, were analyzed by qRT-PCR ( Figure 4A–C). The mRNA expression of *Bcl-2* was downregulated and *Bax* mRNA expression was upregulated in the saline-treated diabetic mice when compared to those in the Ctrl mice. After NMN treatment, *Bcl-2* mRNA expression was significantly increased, while the increase of *Bax* mRNA was reduced. The *Bcl-2*/ *Bax* ratio in the DM group was significantly lower than that in the Ctrl group, and then significantly increased after NMN treatment. Immunohistochemistry of Bcl-2 and Bax protein expressions in testicular cells of saline-treated diabetic mice showed a decrease in the number of Bcl-2 positive cells and an increase in the number of Bax positive cells compared with those in the Ctrl group. Consistently, NMN treatment increased the protein expression level of Bcl-2 and the number of Bcl-2 positive cells, and decreased the protein expression of Bax and the number of Bax positive cells ( Figure 4D–G).

### Effect of NMN on morphology and structure of Sertoli cells in diabetic mice

**Figure 5:**
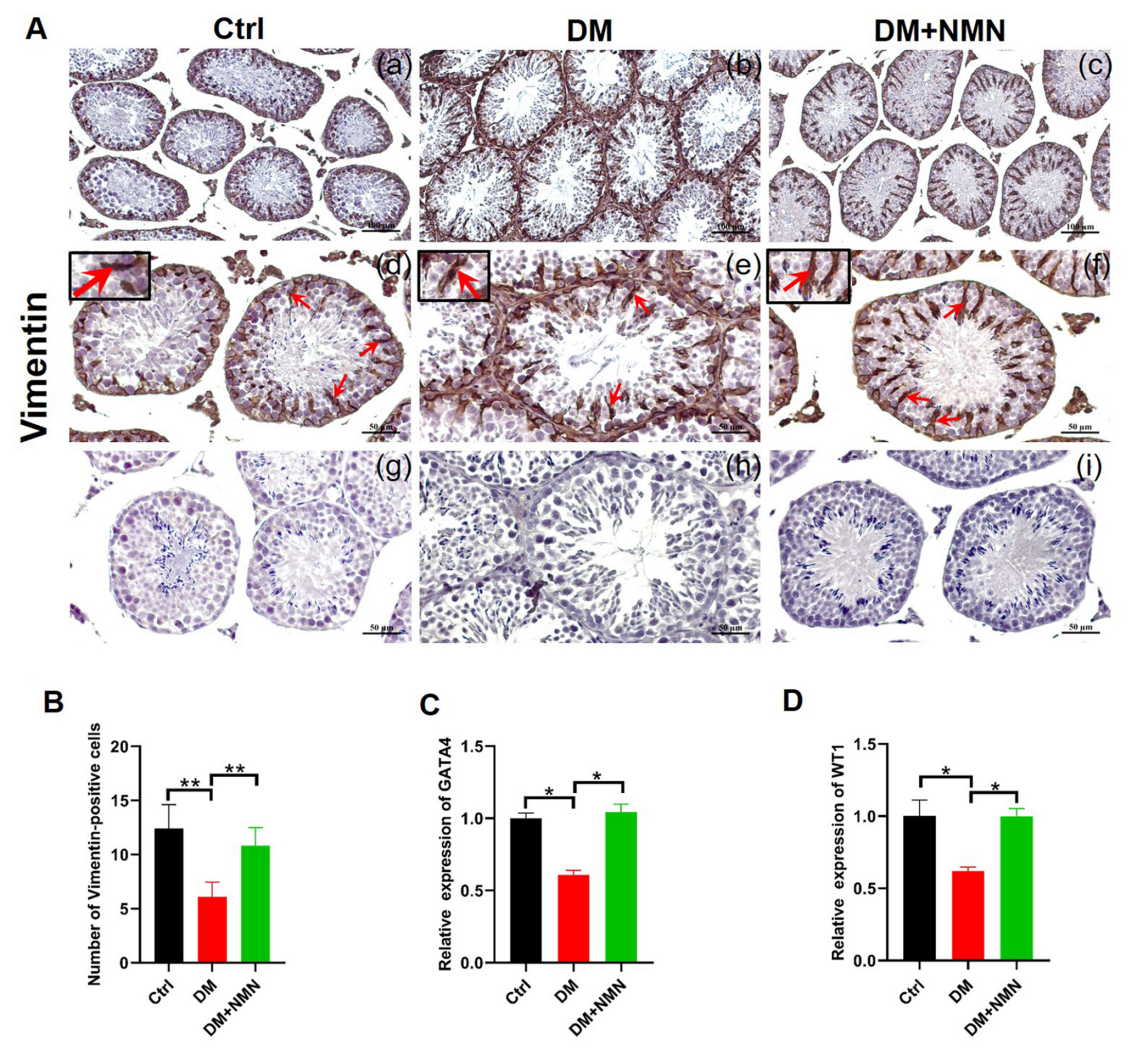
Figure 5 Effect of NMN on the expressions of Sertoli cell markers in diabetic mice (A) Immunohistochemistry analysis of Vimentin expression in testis. The red arrows indicate the cells with positive signals. n=8. Scale bars in (a–c) are 100 μm and in (d–i) are 50 μm. (B) Vimentin positive cell count. (C,D) qRT-PCR analysis of GATA4 and WT1 mRNA expressions in testis. n=4. * P<0.05 and ** P<0.01.

To investigate the expression of Vimentin, an immunomarker of Sertoli cell function and structure, immunohistochemistry analysis was conducted in the testis of the NMN-treated mice. Results demonstrated that Vimentin protein expression in the DM group was scattered and fractured, and the number of Vimentin-positive cells were significantly decreased compared with those in the Ctrl group. After NMN treatment, Vimentin protein expression was similar to the Ctrl group, and the number of Vimentin-positive cells was increased ( Figure 5A,B). Furthermore, the expression levels of Sertoli cell marker genes *GATA4* and *WT1* were down-regulated in the DM group, as determined by qRT-PCR. Conversely, they were up-regulated after NMN treatment ( Figure 5C,D).

### Effect of NMN on testicular glycolysis-related rate-limiting enzymes expression in diabetic mice

**Figure 6:**
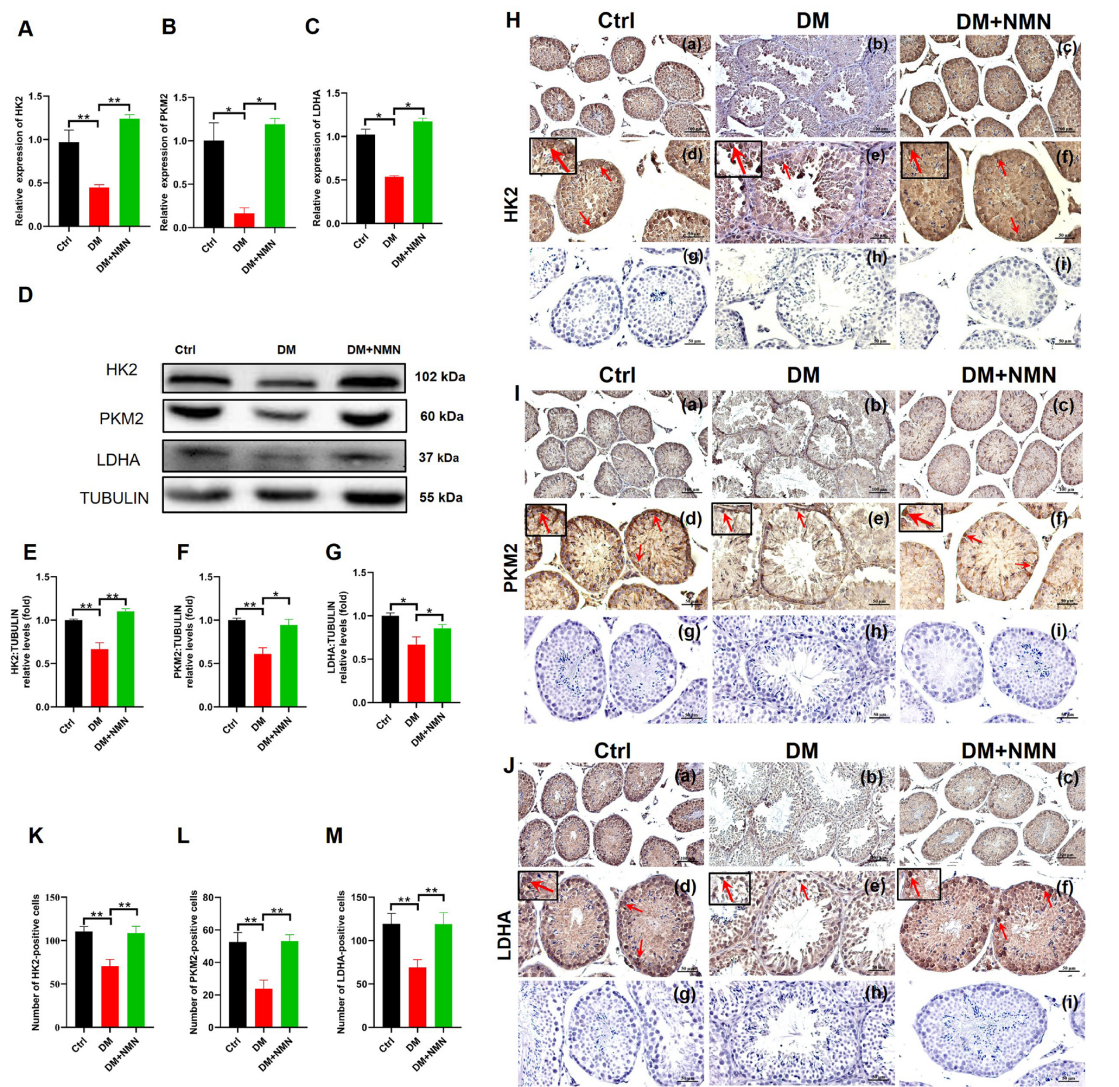
Figure 6 Effect of NMN on the expressions of testicular glycolysis-related rate-limiting enzymes in diabetic mice (A–C) qRT-PCR analysis of HK2, PKM2 and LDHA mRNA expressions in testis. n=4. (D–G) The protein levels of HK2, PKM2, and LDHA were determined by western blot analysis and quantified by ImageJ software. n=4. (H–J) Immunohistochemistry analysis of HK2, PKM2 and LDHA expressions in testis. (K–M) HK2, PKM2 and LDHA positive cell count. The red arrows indicate the cells with a positive signal. Scale bars in (a–c) are 100 μm and in (d–i) are 50 μm. n=8. * P<0.05 and ** P<0.01.

To further investigate the mechanisms of NMN in alleviating spermatogenic cell apoptosis, we examined the expressions of glycolysis-related rate-limiting enzymes by qRT-PCR, western blot analysis, and immunohistochemistry. As shown in Figure 6, the expression levels of *HK2*, *PKM2*, and *LDHA* mRNA in saline-treated diabetic mice were significantly downregulated compared with those in Ctrl mice ( Figure 6A–C). NMN treatment significantly upregulated mRNA expressions of *HK2*, *PKM2*, and *LDHA* in the testicular tissues compared to those in saline-treated diabetic mice. In addition, the diabetes-induced decreases in HK2, PKM2, and LDHA protein levels in mice testis were confirmed by western blot analysis ( Figure 6D–G). Moreover, immunohistochemistry analysis results proved that in the DM group, the protein expressions of HK2, PKM2, and LDHA, as well as the number of positive cells were also decreased ( Figure 6H–M). On the contrary, NMN treatment increased the protein expressions of rate-limiting enzymes related to glycolysis.

## Discussion

DM has become one of the major public health problems of modern societies, as the number of diabetic men during reproductive age has rapidly increased in recent years [7]. Erectile dysfunction and ejaculation difficulties occurred more frequently in diabetic men, ultimately resulting in sexual dysfunction [ 21, 22] . STZ is an antibiotic that could selectively destroy the pancreatic β-cells and is widely used experimentally to produce a DM animal model [23]. Many studies have shown that STZ-induced animals exhibit spermatogenic epithelial arrangement disorders in their testes, accompanied by reduced testis weight and the number of spermatogenic cells and SCs [ 24– 28] . In the present study, we used a single intraperitoneal STZ injection to establish a DM mice model. The results showed decreased sperm quality and testicular spermatogenic dysfunction in the model mice, consistent with previous findings [ 29, 30] . Although numerous medications are available for the treatment of diabetes, various side effects limit their use in clinical practice for diabetic complications. For instance, metformin, a common drug for diabetes, has side effects such as nausea, vomiting, diarrhea, and other gastrointestinal reactions [31]. Therefore, it is of great interest to find effective drugs with effective therapeutic effects on diabetic spermatogenesis disorders. NMN is an NAD ^+^ precursor which has attracted much attention because of its powerful pharmacological effects. NMN has been shown to have beneficial effects to delay aging [32]. However, the effect of NMN on improving diabetic testicular spermatogenic disorder has rarely been reported. In this study, we administered NMN to diabetic mice. The results showed that NMN significantly improved testicular histological injury and sperm parameters, laying a foundation for further exploring the mechanism.

Apoptosis is important for the proper development and maintenance of tissue homeostasis [33], and is recognized as a key event in the pathogenic pathway of DM [34]. Approximately 25%–75% of spermatogenic cells undergo apoptosis during normal spermatogenesis, maintaining the normal reproductive function by regulating the apoptotic signal transduction pathway [35]. Moreover, apoptosis causes depletion of spermatogenic cells in the seminiferous tubules, leading to hollow tubules, spermatogenesis interruption, and infertility [36]. However, hyperglycemia stimulates cell apoptosis and the higher expressions of pro-apoptotic genes [37]. Bcl-2 and Bax are two members of the Bcl-2 family; Bcl-2 inhibits apoptosis, while Bax induces apoptosis [ 38, 39] . The Bcl-2/Bax ratio is a profound indicator of cell survival, reflecting the regulation of apoptosis more clearly than detecting either Bcl-2 or Bax separately [40]. Previous studies have demonstrated that apoptosis in spermatogenic cells is increased in diabetic mice [ 2, 41] , with increased Bax expression and decreased Bcl-2 expression and Bcl-2/Bax ratio [ 42, 43] , resulting in abnormal spermatogenic function. This study also confirmed that the apoptosis of spermatogenic cells in diabetic mice was increased, consistent with the above literatures. In addition, NMN has been reported to suppress oocyte apoptosis by restoring mitochondrial function [44]. Similarly, our data suggest that NMN treatment reduces the spermatogenic cell apoptosis of diabetic mice by significantly upregulating Bcl-2 expression and Bcl-2/Bax ratio.

It is worth noting that SCs are the only somatic cells in the seminiferous epithelium. SCs play a crucial role in the development of testis and spermatogenesis, while their number is usually associated with testis size [45]. *In vitro* experiments have shown that hyperglycemia prevents SC maturation in cultured testicular tissue, which decreases the number of SCs and the area of spermatogenic tubules [46]. Moreover, hyperglycemia exerts a significant adverse effect on SC viability and induces apoptosis in mice [47]. SCs are the main components of the blood-testis barrier, the integrity of which is damaged under high glucose conditions [4]. Vimentin is a SC cytoskeletal protein, and its expression level is directly related to the morphological integrity of seminiferous epithelium [48], as it also affects cell apoptosis and DNA transcription [49]. Wang *et al*. [50] reported that the disordered expression of Vimentin in SCs within cryptorchidism could destroy the function of SCs and increase the spermatogenic cells apoptosis. In addition, it has also been shown that the expression level of Vimentin is down-regulated, which destroys the structure and function of SCs in the testis of diabetic rats [51]. This study verified that Vimentin expression was decreased in the DM mice testis, while NMN supplementation upregulated its expression. As the common marker of the SCs, WT1 can be activated at the early stages of mammalian fetal development and is specifically expressed in the nuclei of SCs throughout all phases of life, while GATA4 is regarded as a specific marker for developing (fetal, postnatal) and adult SCs [52]. Conditional deletion of *GATA4* in SCs of adult mice has been shown to increase the permeability of the blood-testis barrier (BTB), impair lactate production, and disrupt spermatogenesis [53]. Furthermore, WT1 inactivation in adult SCs results in the loss of polarity in SCs and abnormal tight junction assembly, causing spermatogenic cell death [45]. Therefore, it is of great significance to evaluate the function of SCs by detecting the expression levels of WT1 and GATA4. The results of the present study demonstrated for the first time that the expression levels of WT1 and GATA4 were significantly decreased in the DM mice testis, which was restored by NMN supplement. In other words, NMN improves the morphology, structure, and function of SCs in DM mice by upregulating the expressions of Vimentin, WT1, and GATA4.

Moreover, SCs are often the target cells of external toxic substances which cause a direct toxic effect on spermatogenesis by destroying the structure and function of SCs [48]. Furthermore, testes are naturally oxygen-deprived organs. The lactate generated via LDH under SC glycolysis is an important energy source, which could meet the needs of energy and substances for the rapid growth of proliferating spermatogenic cells [ 53, 54] . Three key enzymes, HK2, PKM2, and LDHA, are essential drivers in glycolysis, which increase glucose uptake and lactate production and enhance cell growth [ 55, 56] . Previous studies have shown that diabetes stimulates glycogen accumulation in testis, reduces the energy supply of SCs to spermatocytes and spermatozoa, downregulates the expression level of *LDH* mRNA in SCs, which ultimately increases the number of apoptotic spermatogenic cells [ 57, 58] . In addition, the lactate accumulation in testicular tissue is significantly decreased in diabetic men, concomitant with the reduced expression of LDH protein [59]. Our results showed decreased expressions of HK2, PKM2, and LDHA in the testis of DM mice, including spermatogenic cells and SCs, which are positively correlated with testicular spermatogenic disorder. Some pharmaceutical interventions used for DM currently exhibit better effects for the testicular spermatogenic disorder. Pioglitazone increases the lactate level and stimulates the glycolytic flux in SCs of DM men, significantly improving spermatogenesis dysfunction and male fertility [60]. Moreover, the pharmacological dose of metformin could improve the reproductive potential of diabetic men by decreasing the expression levels of GLUT1, GLUT3, MCT4, and PFK1 and increasing LDH activity, which in turn inhibits apoptosis of spermatogenic cells [61]. In addition, the expressions of GLUT2, GLUT3, PFK1, and the activity of LDH in the testis of prediabetic rats and the improvement of the sperm quality could be elevated by drinking white tea [62]. In the present study, we showed that NMN supplementation could upregulate the expression levels of HK2, PKM2, and LDHA in DM mice testis, indicating that NMN may improve DM-induced spermatogenic dysfunction via regulating the glycolysis pathway. However, the potential mechanisms of NMN in regulating glycolysis pathway need to be further investigated.

In summary, hyperglycemia could damage the structure and function of SCs by inhibiting the glycolysis pathway, leading to apoptosis of spermatogenic cells, and reducing sperm quality. Our results demonstrated that NMN could reverse the changes in body and testis weights, sperm parameters, and testicular histology in STZ-induced diabetic mice. Furthermore, NMN also reduces spermatogenic cells apoptosis, upregulates the expressions of SC markers and glycolytic-related rate-limiting enzymes in testes. Therefore, our findings suggest that NMN may positively affect spermatogenic dysfunction by promoting the glycolysis pathway, providing a potential target for DM treatment with NMN.

## References

[1] Kocaman N, Kuloğlu T (2020). Expression of asprosin in rat hepatic, renal, heart, gastric, testicular and brain tissues and its changes in a streptozotocin-induced diabetes mellitus model. Tissue Cell.

[2] Liu Y, Yang Z, Kong D, Zhang Y, Yu W, Zha W (2019). Metformin ameliorates testicular damage in male mice with streptozotocin-induced type 1 diabetes through the PK2/PKR pathway. Oxid Med Cell Longev.

[3] Long L, Qiu H, Cai B, Chen N, Lu X, Zheng S, Ye X (2018). Hyperglycemia induced testicular damage in type 2 diabetes mellitus rats exhibiting microcirculation impairments associated with vascular endothelial growth factor decreased via PI3K/Akt pathway. Oncotarget.

[4] Jiang YP, Ye RJ, Yang JM, Liu N, Zhang WJ, Ma L, Sun T (2020). Protective effects of Salidroside on spermatogenesis in streptozotocin induced type-1 diabetic male mice by inhibiting oxidative stress mediated blood-testis barrier damage. Chemico-Biol Interactions.

[5] Ding GL, Liu Y, Liu ME, Pan JX, Guo MX, Sheng JZ, Huang HF (2015). The effects of diabetes on male fertility and epigenetic regulation during spermatogenesis. Asian J Androl.

[6] Mao CF, Zhang XR, Johnson A, He JL, Kong ZL (2018). Modulation of diabetes mellitus-induced male rat reproductive dysfunction with micro-nanoencapsulated
*Echinacea purpurea* ethanol extract. Biomed Res Int.

[7] Alves MG, Martins AD, Rato L, Moreira PI, Socorro S, Oliveira PF (2013). Molecular mechanisms beyond glucose transport in diabetes-related male infertility. Biochim Biophys Acta (BBA) - Mol Basis Dis.

[8] Yi X D, Zhang Y N, Xiao S, Lei X C. Role and regulatory mechanism of glycometabolism of sertoli cells in spermatogenesis.
*
National Journal of Andrology Zhonghua Nan Ke Xue Za Zhi
* 2019, 25: 923–927. https://pubmed.ncbi.nlm.nih.gov/32233225/.

[9] Yoshino J, Baur JA, Imai SI (2018). NAD+ intermediates: the biology and therapeutic potential of NMN and NR. Cell Metab.

[10] Wang X, Hu X, Yang Y, Takata T, Sakurai T (2016). Nicotinamide mononucleotide protects against β-amyloid oligomer-induced cognitive impairment and neuronal death. Brain Res.

[11] Hong W, Mo F, Zhang Z, Huang M, Wei X (2020). Nicotinamide mononucleotide: A promising molecule for therapy of diverse diseases by targeting NAD+ metabolism. Front Cell Dev Biol.

[12] North BJ, Rosenberg MA, Jeganathan KB, Hafner AV, Michan S, Dai J, Baker DJ (2014). SIRT2 induces the checkpoint kinase BubR1 to increase lifespan. EMBO J.

[13] Guan Y, Wang SR, Huang XZ, Xie QH, Xu YY, Shang D, Hao CM (2017). Nicotinamide mononucleotide, an NAD
^+^ precursor, rescues age-associated susceptibility to AKI in a sirtuin 1–dependent manner. J Am Soc Nephrol.

[14] Poddar SK, Sifat AE, Haque S, Nahid NA, Chowdhury S, Mehedi I (2019). Nicotinamide mononucleotide: exploration of diverse therapeutic applications of a potential molecule. Biomolecules.

[15] Revollo JR, Körner A, Mills KF, Satoh A, Wang T, Garten A, Dasgupta B (2007). Nampt/PBEF/visfatin regulates insulin secretion in β cells as a systemic NAD biosynthetic enzyme. Cell Metab.

[16] Ramsey KM, Mills KF, Satoh A, Imai SI (2008). Age-associated loss of Sirt1-mediated enhancement of glucose-stimulated insulin secretion in beta cell-specific Sirt1-overexpressing (BESTO) mice. Aging Cell.

[17] Mills KF, Yoshida S, Stein LR, Grozio A, Kubota S, Sasaki Y, Redpath P (2016). Long-term administration of nicotinamide mononucleotide mitigates age-associated physiological decline in mice. Cell Metab.

[18] Benjamin D, Robay D, Hindupur SK, Pohlmann J, Colombi M, El-Shemerly MY, Maira SM (2018). Dual inhibition of the lactate transporters MCT1 and MCT4 is synthetic lethal with metformin due to NAD+ depletion in cancer cells. Cell Rep.

[19] Rabinowitz JD, Enerbäck S (2020). Lactate: the ugly duckling of energy metabolism. Nat Metab.

[20] Li Z, Wang C, Song D Q, Jiang J D, Kong W J. Study of anti-diabetic nephropathy efficacy of berberine analogue Y53 in STZ-induced diabetic C57BL/6J mice.
*
Chinese Pharmacological Bulletin
* 2016, 32: 1236–1242. https://doi.org/10.3969/j.issn.1001-1978.2016.09.010.

[21] Tamas V, Kempler P. Sexual dysfunction in diabetes.
*
Handb Clin Neurol
* 2014, 126: 223–232. https://doi.org/10.1016/B978-0-444-53480-4.00017-5.

[22] Maiorino MI, Bellastella G, Esposito K (2014). Diabetes and sexual dysfunction: current perspectives. DMSO.

[23] Furman BL (2015). Streptozotocin‐induced diabetic models in mice and rats. Curr Protocols Pharmacol.

[24] Roshankhah S, Jalili C, Salahshoor MR (2019). Effects of crocin on sperm parameters and seminiferous tubules in diabetic rats. Adv Biomed Res.

[25] Rahimiyan-Heravan M, Roshangar L, Karimi P, Sefidgari-Abrasi S, Morshedi M, Saghafi-Asl M, Bavafa-Valenlia K (2020). The potential therapeutic effects of Lactobacillus plantarum and inulin on serum and testicular reproductive markers in diabetic male rats. Diabetol Metab Syndr.

[26] Jiang SJ, Dong H, Fang K, Chen G, Li JB, Xu LJ, Zou X (2021). Protective effects of hu-lu-ba-wan (葫芦巴丸) against oxidative stress in testis of diabetic rats through PKCα/NAPDH oxidase signaling pathway. Chin J Integr Med.

[27] Zhou J, Xi Y, Zhang J, Tang J, Zhou X, Chen J, Nie C (2020). Protective effect of
*Dioscorea zingiberensis* ethanol extract on the disruption of blood–testes barrier in high‐fat diet/streptozotocin‐induced diabetic mice by upregulating ZO‐1 and Nrf2. Andrologia.

[28] Shoorei H, Khaki A, Shokoohi M, Khaki AA, Alihemmati A, Moghimian M, Abtahi-Eivary SH (2020). Evaluation of carvacrol on pituitary and sexual hormones and their receptors in the testicle of male diabetic rats. Hum Exp Toxicol.

[29] Abdullah F, Khan Nor-Ashikin MN, Agarwal R, Kamsani YS, Abd Malek M, Bakar NS, Mohammad Kamal AA (2021). Glutathione (GSH) improves sperm quality and testicular morphology in streptozotocin-induced diabetic mice. Asian J Androl.

[30] Fajri M, Ahmadi A, Sadrkhanlou R (2020). Protective effects of equisetum arvense methanolic extract on sperm characteristics and in vitro fertilization potential in experimental diabetic mice: an experimental study. IJRM.

[31] Lei X, Huo P, Wang Y, Xie Y, Shi Q, Tu H, Yao J (2020). Lycium barbarum polysaccharides improve testicular spermatogenic function in streptozotocin-induced diabetic rats. Front Endocrinol.

[32] Min H, Lee M, Cho KS, Lim HJ, Shim YH (2021). Nicotinamide supplementation improves oocyte quality and offspring development by modulating mitochondrial function in an aged caenorhabditis elegans model. Antioxidants.

[33] Singh R, Letai A, Sarosiek K (2019). Regulation of apoptosis in health and disease: the balancing act of BCL-2 family proteins. Nat Rev Mol Cell Biol.

[34] Samaha MM, Said E, Salem HA (2019). A comparative study of the role of crocin and sitagliptin in attenuation of STZ-induced diabetes mellitus and the associated inflammatory and apoptotic changes in pancreatic β-islets. Environ Toxicol Pharmacol.

[35] Ning JZ, Rao T, Cheng F, Yu WM, Ruan Y, Yuan R, Zhu SM (2017). Effect of varicocelectomy treatment on spermatogenesis and apoptosis via the induction of heat shock protein 70 in varicocele-induced rats. Mol Med Rep.

[36] Wang J, Gao WJ, Deng SL, Liu X, Jia H, Ma WZ (2019). High temperature suppressed SSC self-renewal through S phase cell cycle arrest but not apoptosis. Stem Cell Res Ther.

[37] Zhang Z, Liew CW, Handy DE, Zhang Y, Leopold JA, Hu J, Guo L (2010). High glucose inhibits glucose‐6‐phosphate dehydrogenase, leading to increased oxidative stress and β‐cell apoptosis. FASEB J.

[38] Mohammadpour-Gharehbagh A, Jahantigh D, Eskandari M, Sadegh MH, Nematollahi MH, Rezaei M, Rasouli A (2019). Genetic and epigenetic analysis of the BAX and BCL2 in the placenta of pregnant women complicated by preeclampsia. Apoptosis.

[39] He W, Liu H, Hu L, Wang Y, Huang L, Liang A, Wang X (2021). Icariin improves testicular dysfunction via enhancing proliferation and inhibiting mitochondria-dependent apoptosis pathway in high-fat diet and streptozotocin-induced diabetic rats. Reprod Biol Endocrinol.

[40] Tian W, Heo S, Kim DW, Kim IS, Ahn D, Tae HJ, Kim MK (2021). Ethanol extract of maclura tricuspidata fruit protects SH-SY5Y neuroblastoma cells against H
_2_O
_2_-induced oxidative damage via inhibiting MAPK and NF-κB signaling. Int J Mol Sci.

[41] Jiang X, Bai Y, Zhang Z, Xin Y, Cai L (2014). Protection by sulforaphane from type 1 diabetes-induced testicular apoptosis is associated with the up-regulation of Nrf2 expression and function. Toxicol Appl Pharmacol.

[42] Caiaffo1 V, Oliveira B D R. Diabetes mellitus, testicular damages and seafood: a curious relationship.
*
Current Diabetes Reviews
* 2016: 1–7. https://www.semanticscholar.org/paper/Diabetes-Mellitus%2C-Testicular-Damages-and-Seafood%3A-Caiaffo-Bd/d1e682889e3c6321282ef7e75283bb65ee61a9a2.

[43] Zhao Y, Tan Y, Dai J, Li B, Guo L, Cui J, Wang G (2011). Exacerbation of diabetes-induced testicular apoptosis by zinc deficiency is most likely associated with oxidative stress, p38 MAPK activation, and p53 activation in mice. Toxicol Lett.

[44] Miao Y, Cui Z, Gao Q, Rui R, Xiong B (2020). Nicotinamide mononucleotide supplementation reverses the declining quality of maternally aged oocytes. Cell Rep.

[45] Wang XN, Li ZS, Ren Y, Jiang T, Wang YQ, Chen M, Zhang J (2013). The Wilms tumor gene, Wt1, is critical for mouse spermatogenesis via regulation of sertoli cell polarity and is associated with non-obstructive azoospermia in humans. PLoS Genet.

[46] Tavares RS, Portela JMD, Sousa MI, Mota PC, Ramalho-Santos J, Amaral S (2017). High glucose levels affect spermatogenesis: an in vitro approach. Reprod Fertil Dev.

[47] Gan DM, Zhang PP, Zhang JP, Ding SX, Fang J, Liu Y (2021). KISS1/KISS1R mediates sertoli cell apoptosis via the PI3K/AKT signalling pathway in a high‑glucose environment. Mol Med Rep.

[48] Vaz AC, Paccola CC, Mendes TB, Cabral REL, Simas JN, Vendramini V, Miraglia SM (2020). Sertoli cell alterations in peripubertal varicocelized rats: evidence of primary damage on spermatogenesis. J Histochem Cytochem.

[49] Mohamed H, Haglund C, Jouhi L, Atula T, Hagström J, Mäkitie A (2020). Expression and role of e-cadherin, β-catenin, and vimentin in human papillomavirus–positive and human papillomavirus–negative oropharyngeal squamous cell carcinoma. J Histochem Cytochem.

[50] Wang ZQ, Watanabe Y, Toki A, Itano T (2002). Altered distribution of Sertoli cell vimentin and increased apoptosis in cryptorchid rats. J Pediatr Surg.

[51] Xu Y, Lei H, Guan R, Gao Z, Li H, Wang L, Hui Y (2014). Prophylactic protective effects and its potential mechanisms of icarisideII on streptozotocin induced spermatogenic dysfunction. Int J Mol Sci.

[52] You X, Chen Q, Yuan D, Zhang C, Zhao H (2021). Common markers of testicular Sertoli cells. Expert Rev Mol Diagnostics.

[53] Schrade A, Kyrönlahti A, Akinrinade O, Pihlajoki M, Fischer S, Rodriguez VM, Otte K (2016). GATA4 regulates blood-testis barrier function and lactate metabolism in mouse sertoli cells. Endocrinology.

[54] Odet F, Gabel SA, Williams J, London RE, Goldberg E, Eddy EM (2011). Lactate dehydrogenase C and energy metabolism in mouse sperm. Biol Reprod.

[55] Nakatsu D, Horiuchi Y, Kano F, Noguchi Y, Sugawara T, Takamoto I, Kubota N (2015). L-cysteine reversibly inhibits glucose-induced biphasic insulin secretion and ATP production by inactivating PKM2. Proc Natl Acad Sci USA.

[56] Yin D, Hua L, Wang J, Liu Y, Li X (2020). Long non-coding RNA DUXAP8 facilitates cell viability, migration, and glycolysis in non-small-cell lung cancer via regulating HK2 and LDHA by inhibition of miR-409-3p. OTT.

[57] Alves MG, Martins AD, Cavaco JE, Socorro S, Oliveira PF (2014). Diabetes, insulin-mediated glucose metabolism and Sertoli/blood-testis barrier function. Tissue Barriers.

[58] Rato L, Alves MG, Dias TR, Cavaco JE, Oliveira PF (2015). Testicular metabolic reprogramming in neonatal streptozotocin-induced type 2 diabetic rats impairs glycolytic flux and promotes glycogen synthesis. J Diabetes Res.

[59] Alves MG, Martins AD, Moreira PI, Carvalho RA, Sousa M, Barros A, Silva J (2015). Metabolic fingerprints in testicular biopsies from type 1 diabetic patients. Cell Tissue Res.

[60] Meneses MJ, Bernardino RL, Sá R, Silva J, Barros A, Sousa M, Silva BM (2016). Pioglitazone increases the glycolytic efficiency of human Sertoli cells with possible implications for spermatogenesis. Int J Biochem Cell Biol.

[61] Alves MG, Martins AD, Vaz CV, Correia S, Moreira PI, Oliveira PF, Socorro S (2014). Metformin and male reproduction: effects on Sertoli cell metabolism. Br J Pharmacol.

[62] Dias TR, Alves MG, Rato L, Casal S, Silva BM, Oliveira PF (2016). White tea intake prevents prediabetes-induced metabolic dysfunctions in testis and epididymis preserving sperm quality. J Nutral Biochem.

